# The hidden inequity in health care

**DOI:** 10.1186/1475-9276-10-15

**Published:** 2011-04-20

**Authors:** Barbara Starfield

**Affiliations:** 1 University Distinguished Professor, Department of Health Policy and Management, Johns Hopkins University, 624 North Broadway, room 452, Baltimore, MD 21205, USA

## 

Inequity is the presence of systematic and potentially remediable differences among population groups defined socially, economically, or geographically [1,2]. It is not the same as inequality, which is a much broader term, generally used in the human rights field to describe differences among individuals, some of which are not remediable (at least with current knowledge). Some languages do not make a distinction between the two terms, which may lead to confusion and a need to clarify exact meaning in different contexts. Some people use the term "unfairness" to define inequity, but unfairness is not measurable and therefore not a useful term for policy or evaluation.

Inequity can be horizontal or vertical. Horizontal inequity indicates that people with the same needs do not have access to the same resources. Vertical inequity exists when people with greater needs are not provided with greater resources. In population surveys, similar use of services across population groups signifies inequity, because different population subgroups have different needs, some more than others. What is generally considered equity (equal use across population subgroups) is, in fact, inequity.

Most industrialized countries have achieved both horizontal and vertical equity in the use of primary care services, meaning that people with greater health needs receive more primary care services. Although some countries have achieved horizontal equity in use of specialist services, very few have achieved vertical equity because socially-deprived populations have less access to specialist services than their needs require.

There are no statistics on inequity in health in different countries. All standard health statistics describe average or "mean" health in the population - life expectancy, infant mortality, death rates from various diseases, and the like. Health indicators that are used to describe various aspects of population health and the impact of services on them are also useful for assessing equity in health. Producing them only requires stratifying the population into the social, economic, or geographic indicator and determining if there are differences in the rates of the indicator across the strata. As equity is an international priority, countries should be collecting data on inequities among groups in the population.

Although equity in use of services is a worldwide imperative, an even more serious challenge is posed by the way of thinking about illness and its impact. The very underpinnings of modern-day health services are inequitable.

Western health systems are dominated by a paradigm of illness that considers "diseases" to be the basic element of pathology [3]. Beginning with the anatomist Vesalius in the 17^th ^century, disease came to be thought of in terms of abnormalities in body organs, with each abnormality adding, in linear fashion, to the extent of illness. Medicine is still practiced this way, with each disease requiring special knowledge and special expertise for management, and adherence to each disease guideline adding linearly to the quality of care provided. In this outdated scheme, there is no room for recognizing that diseases are not distinct biological entities that exist alone and apart from the person. A century ago, thoughtful clinicians (such as Sir William Osler) recognized that it is more important to know "what sort of patient has a disease than to know what sort of disease a patient has" [4]. The only change that might be made to this dictum a century later is to substitute diseases, risk factors, and adverse effects for "disease".

A "whole-patient oriented" view of disease is more accurate than a disease oriented view. It is also more equitable. Diseases are more likely to occur and to be more serious in socially disadvantaged people [2,5]. This greater likelihood of occurrence, severity, and adverse effects is compounded even further by multiple illnesses, multiple serious illnesses, and greater likelihood of adverse events from incompatible interventions. Only a person-focused (rather than a disease-focused) view of morbidity, in which multiple illnesses interact in myriad ways, can accurately depict the much greater impact of illness among socially disadvantaged people and the nature of the interventions that are required to adequately manage the increased vulnerability to and interactions among diseases.

The historical development of health statistics, based on coroners' reports of anatomical pathology noticed on autopsies, followed from the view of illness as separate and distinct pathologies. Thus, right from the beginning, health statistics were collected body system by system, thus providing the basis for modern medical education by organ system specialties: cardiologists, pulmonologists, urologists, vascular surgeons - and so on. Organ system based medicine is becoming dysfunctional, because most illness nowadays is multimorbidity - cutting across diseases and types of diseases and organ systems. But information on health problems is collected disease by disease. Doing so masks the greater needs of people in different population subgroups, because they are more vulnerable to and suffer more different types of illnesses and combinations of illness. Disease-oriented medicine, whether through guidelines or through a focus on particular chronic diseases and their management is thus highly inequitable as it cannot address the adequacy of interventions when people have many problems.

Inequity is built into health systems - especially western health systems that are based on a view of health needs disease-by disease. Therefore, the benefits of primary care, which is person- and population- rather than disease-focused, are underappreciated. Data provide evidence not only of its benefit to populations but also of its preferential benefit to the socially disadvantaged [6]. Increasing referral rates from primary care to specialty care pose a special problem for socially deprived population groups, as their greater morbidity leads them to be referred to more different types of specialists with consequent increased likelihood of poor coordination, adverse effects, and unnecessarily high costs (some which will come from out-of-pocket payments) unless there is strong primary care. Disease specialists are unable to deal with interactions among types of diseases; their utility is primarily for advice or intervention for time-limited events (either diagnostic or therapeutic) occurring in the course of illness. Primary care must inevitably assume increasing importance in health systems because it is far superior in dealing with multimorbidity over time. This is part of the explanation for its greater contribution to health in modern societies.

What makes certain people and certain populations costly is not that they have more chronic disease. It is that they have more types of morbidity [7,8] (Shadmi E, Balicer RD, Kinder K, Abrams C, Starfield B, Weiner JP: Morbidity and health care resource use: beyond chronic condition counts, submitted). Over the past few years, there have been elegant studies that show that when populations are characterized according to their morbidity burdens, the greater costs of care are NOT a result of their having costly chronic disease. It is because they are vulnerable to and have more different types of illnesses. Diseases are not unique entities; there are greater differences in resource needs within disease categories than across them. Diseases do not exist in isolation; having one disease predisposes to others [9]. People have health problems but diseases are only a partial explanation for their health problems. The problems that bother and disable people, such as chronic pain, deserve more attention because many of these problems cannot be related to specific diseases. We need to know what health problems people suffer, quite apart from what diagnostic label is attached to them by health professionals. Problem improvement and problem resolution (and, conversely, worsening, which is often a result of adverse medical effects) are legitimate measures of outcome, and practitioners who are better at recognizing problems and dealing with them should be rewarded for doing so. We need guidelines that are appropriate to person-focused care, not disease focused care. Only primary care physicians can understand this, because they do not focus on particular organ systems and because they experience these realities every day in their practices. Primary care physicians and, especially, family physicians will have to continue to advocate for primary care-oriented health systems, because it is the only hope for achieving greater equity through appropriate medical interventions. They have an even greater responsibility, however, and that is to draw attention to the folly of providing care disease-by-disease. There are systems to characterize people according to their morbidity burden - various combinations of different types of illnesses. All health systems that have electronic capability should be collecting data in a way that enables the calculation of morbidity burden from data on separate diseases; the technology to do so is available.

It is time that primary care physicians take leadership in moving medical care where it needs to be: to the care of patients and populations and not the care of diseases. It is not only biologically correct to do so - it is also more effective, more efficient, safer, and more equitable.
